# 20 years of communicating facts and figures

**DOI:** 10.2807/1560-7917.ES.2016.21.48.30415

**Published:** 2016-12-01

**Authors:** Ines Steffens

**Affiliations:** 1 European Centre for Disease Prevention and Control (ECDC), Stockholm, Sweden

Since 1995, when a first pilot issue was published, *Eurosurveillance* has provided the European public health community with a platform to exchange relevant findings on communicable disease surveillance, prevention and control. From the outset, the journal has been open access and has not charged article processing costs.

In 2016, we celebrate 20 years of regular publication. A glimpse at the *Eurosurveillance* archives demonstrates how the journal has matured over the years in terms of format and content. It shows, for example, the merging of the formerly weekly and monthly issues, acceptance of the ‘weekly’ for indexing in PubMed/MEDLINE and the evolution from a print and online journal to a full online journal and a gradual geographical expansion of the origin of published articles.

However, already from the start, topics covered were remarkably similar to those that are high on the public health agenda today. One of the articles in the pilot issue in 1995 gave an overview of immunisation schedules in Europe [1], a topic still of interest nowadays. Our aim to provide insightful and balanced information on vaccination was shown after the later retracted publication by Wakefield et al. that included subsequently falsified claims of an association of measles mumps and rubella vaccines with autism [2]. Just one week afterwards, *Eurosurveillance* ran a commentary in its weekly edition, followed, two months later, by one entitled ‘Further evidence that MMR vaccine, inflammatory bowel disease, and autism are not linked’ [3,4]. The public health challenges that Europe faces in reaching the measles elimination goal in Europe were marked in a ‘Spotlight on measles’ series on ongoing outbreaks and their implications [5].

Since the early days of the journal, surveillance network outputs and outbreak reports have been regular content [6], with topics such as HIV/AIDS and other sexually transmitted infections [7-9], emerging (vector-borne) diseases [10], influenza [11], antimicrobial resistance [12], tuberculosis [13,14] and food- and waterborne diseases [15]. As illustrated by the following subjective selection of articles from the past two decades, public health events and other topics with general public health relevance have also been covered, such as the preparedness for bioterrorism after the 11 September attacks in 2001 in the United States [16], the outbreak of severe acute respiratory syndrome (SARS) [17], the 2009 influenza pandemic [18], the emergence of Middle East respiratory syndrome (MERS) [19], as well as the setup of the European Programme for Intervention Epidemiology Training (EPIET) programme [20] and discussions about establishing a European Centre for Disease Control [21].

Rapid communications were an early feature for the journal at a time when rapid processing of articles was not a common element of scientific journals. The evolution, growth and opportunities offered by the Internet facilitated timely communication and fast turnaround times tremendously. The initially short news-like items are the element of the journal that has most evolved. Today, rapid communications are well-recognised short scientific dispatches. Several of them are among our most highly cited articles, but more importantly, their value has been in their impact on public health practice.

While we have been able to present ‘firsts’ on several occasions [22,23] and track epidemics and emerging diseases in a timely manner [24], we are publishing an increasing number of (systematic) reviews to provide sound evidence and support for decisionmaking [25].

Working with *Eurosurveillance* is rewarding. The journal has many supporters and collaborators in Europe and beyond whom we are not able to name individually. We would like to express our gratitude to them and also thank our board members, colleagues and publisher wholeheartedly for their continued support. Our 20th anniversary is a reason to celebrate. We marked the occasion on Wednesday 30 November with a lunchtime seminar ‘20 years of communicating facts and figures in a changing environment’, held on the margins of the European Scientific Conference on Applied Infectious Diseases Epidemiology (ESCAIDE). Two eminent speakers, David Heymann and Lawrence Madoff, highlighted changes in sharing information about communicable diseases from a public health perspective over the past 20 years. In addition, we present this selection of articles as a snapshot of the journal’s publications and evolution. The topics covered match those that have remained relevant over two decades and we hope our readers will enjoy browsing through this compilation.
